# The Association of a Mediterranean-Style Diet Pattern with Polycystic Ovary Syndrome Status in a Community Cohort Study

**DOI:** 10.3390/nu7105419

**Published:** 2015-10-16

**Authors:** Lisa J. Moran, Jessica A. Grieger, Gita D. Mishra, Helena J. Teede

**Affiliations:** 1 The Robinson Research Institute, University of Adelaide, 55 King William Road, North Adelaide, South Australia 5006, Australia; jessica.grieger@adelaide.edu.au; 2 Monash Centre for Health Research Implementation, School of Public Health and Preventative Medicine, Monash University, Melbourne 3004, Australia; helena.teede@monash.edu; 3 School of Public Health, University of Queensland, Herston, Queensland 4006, Australia; g.mishra@sph.uq.edu.au; 4 Diabetes and Endocrine Unit, Monash Health, Clayton 3168, Australia

**Keywords:** polycystic ovary syndrome, diet, dietary patterns, Australia

## Abstract

Polycystic ovary syndrome (PCOS) is a common condition in reproductive-aged women. While lifestyle management is first-line treatment in PCOS, the dietary intake of women with PCOS is unclear and there is no research assessing dietary patterns of women with and without PCOS. The aim of this study was to examine dietary patterns in a large cohort of women with and without PCOS. Data were from 7569 participants in the 1973–1978 cohort of the Australian Longitudinal Study on Women’s Health population assessed at 2009 (Survey 5) (*n* = 414 PCOS, *n* = 7155 non-PCOS). Dietary patterns were evaluated using factor analysis and multiple logistic regressions assessed their associations with PCOS status. Three dietary patterns were identified that explained 27% of the variance in food intake between women with and without PCOS: Non-core foods; Meats and take-away and Mediterranean-style. The Mediterranean-style dietary pattern was independently associated with PCOS status. On adjusted analysis for each 1 SD increase in the Mediterranean-style dietary pattern, there was a 26% greater likelihood that women had PCOS. This may indicate an improvement in the quality of dietary intake following a diagnosis of PCOS. Future research should examine the contribution of dietary patterns to the incidence and severity of PCOS and the potential for modification of dietary patterns in the lifestyle management of PCOS.

## 1. Introduction

Polycystic ovary syndrome (PCOS) is a common endocrine condition affecting 12%–18% of reproductive-aged women [1]. It is associated with reproductive (hyperandrogenism, anovulation, menstrual irregularly, infertility and pregnancy complications) [2], metabolic (increased risk factors for and prevalence of impaired glucose tolerance, type 2 diabetes and cardiovascular disease) [3,4,5] and psychological (worsened quality of life and increased risk factors for depression and anxiety) [6] features. There is a proposed bidirectional relationship between obesity and PCOS [7]. Women with PCOS have an elevated prevalence of obesity [8] and increased longitudinal weight gain [7]. Obesity also worsens the presentation and prevalence of PCOS [9]. Mechanisms include increasing the pathophysiological factor insulin resistance, which increases hyperandrogenism through augmenting ovarian androgen production and decreasing hepatic production of the androgen binding-protein sex hormone binding globulin [10,11]. Due to the key aetiological role of obesity and insulin resistance in PCOS, weight management, defined as prevention of excess weight gain or achieving and maintaining a modest weight loss, is a key treatment strategy in PCOS. Evidence based guidelines recommend achieving this through a combination of diet, exercise or behavioural management [12].

The optimal dietary strategy as part of lifestyle management in PCOS remains controversial. We reported in a recent systematic review that the controlled clinical literature found no difference in the majority of anthropometric, reproductive, metabolic or psychological outcomes for a range of dietary approaches including higher protein, higher carbohydrate, lower glycaemic index or monounsaturated fat-enriched diets [13]. Despite this, a range of dietary approaches may be prescribed by health professionals [14]. While evidence-based National Health and Medical Council approved Australian guidelines outline the principles of dietary management for PCOS [12], the effect of these guidelines on actual dietary prescription by health professionals and subsequent dietary intake by women with PCOS is not known. In the absence of specific recommendations by health professionals, women with PCOS may also often seek non-evidence based sources of information on dietary management [15]. The effect of this on actual dietary intake is not known. We and others have reported subtle differences in dietary intake for women with PCOS compared to those without PCOS including a better dietary intake as indicated by elevated diet quality indices, fibre and micronutrient intake, lower glycaemic index and lower total fat or saturated fat intake or a poorer dietary intake indicated by poorer diet quality, increased fat, saturated fat and high glycaemic index food intake and decreased fibre intake compared to women without PCOS [16,17,18,19,20,21]. There however remains uncertainty as to the quality of dietary intake in women with PCOS.

Assessment of dietary patterns offers an additional way of comprehensively assessing dietary intake. Rather than assessing single nutrients in isolation, dietary pattern analysis identifies underlying dietary characteristics of the study population in which the consumption of foods that are eaten together can be derived. In particular, exploratory approaches or posteriori dietary pattern analyses such as principal components analysis, which are not hypothesis driven, groups correlated food groups into uncorrelated factors termed dietary patterns [22,23]. In pregnant populations, unhealthy dietary patterns in the pre-conception period were associated with increased risk for preterm birth [24] or gestational diabetes [25] and healthy, Mediterranean or prudent diet patterns were inversely associated with risk of developing hypertensive disorders during pregnancy [26] or gestational diabetes [27,28]. In non-pregnant populations, unhealthy/Western-type dietary patterns have been associated with increased risk of general and central obesity [29] and type 2 diabetes [30]; while a Mediterranean dietary pattern was associated with decreased prevalence of hypertension and metabolic syndrome [31] and a healthy dietary pattern containing vegetables, fruits and whole grains was associated with reduced risk for diabetes [30].

These findings are of potential relevance to PCOS given the increased prevalence of cardiometabolic conditions and pregnancy complications and the potential for clinical benefits with approaches such as the Mediterranean diet [32]. However, there has been limited research examining dietary patterns in women with and without PCOS. This could provide an understanding on both the association of dietary intake with the pathophysiology of PCOS as well as of the dietary changes that occur following a diagnosis of PCOS. The aim of this study was therefore to examine dietary patterns in a large cohort of women, with and without PCOS, participating in the Australian Longitudinal Study on Women’s Health.

## 2. Experimental Section

### 2.1. Study Population

This study is based on data from the Australian Longitudinal Study on Women’s Health (ALSWH), a longitudinal population-based study of three age cohorts of Australia women. Women were randomly selected from the national health insurance scheme (Medicare) database, which includes almost all people who are permanent residents of Australia, with national recruitment and intentional over-sampling from rural and remote areas [33]. Further details of the methods and characteristics of the sample have been reported elsewhere [34,35,36]. The Human Research Ethics Committees of the University of Newcastle and the University of Queensland approved the study methods and informed written consent was obtained from each participant.

The current study uses data from the cohort of younger women (born 1973–1978) (*n* = 14,779 at Survey 1) who first completed a mailed survey in 1996 [37]. For this analysis, data are from Survey 5 (2009, *n* = 8200, 58% retention of baseline participants and 84% retention of those who completed the second survey). The greatest drop out was from Survey 1 to Survey 2. However, the impact of attrition on associations between variables has been found to be minimal [36].

As with our prior publications on health outcomes in PCOS, we analysed data from *n* = 7569 women who completed Survey 5 and responded to the question on PCOS diagnosis (“In the last 3 years have you been diagnosed with or treated for Polycystic Ovary Syndrome”) of which *n* = 414 were classified as PCOS and *n* = 7155 as non-PCOS [7]. The analyses in this study are based on cross-sectional analysis of dietary patterns in women with and without PCOS. No specific inclusion or exclusion criteria were applied to this cohort and all women were included irrespective of pregnancy, medication, country of birth and language spoken.

### 2.2. Anthropometric, Demographic and Physical Activity Variables

Self-reported height, weight and BMI were reported with overweight and obesity defined by the World Health Organization criteria (BMI ≥ 25 kg/m^2^ for overweight and obesity, BMI ≥ 30 kg/m^2^ for obesity) [38]. Demographic variables including parity, education, occupation and income were collected at Survey 5 and area of residence was measured at Survey 1. Physical activity was calculated as the sum of the products of total weekly minutes in categories of walking, moderate-intensity or vigorous-intensity physical activity and the metabolic equivalent value (MET) was assigned to each category: (walking minutes × 3.0 METs) + (moderate-intensity physical activity minutes × 4.0 METs) + (vigorous intensity physical activity minutes × 7.5 METs). Outliers were truncated at 28 h/day for total physical activity.

### 2.3. Food Group Consumption

At Survey 5, self-reported dietary intake data were collected from the Dietary Questionnaire for Epidemiological Studies (DQES) Version 2, a FFQ developed by The Cancer Council of Victoria previously validated in young Australian women [39] as previously reported [19]. One hundred different foods (grams per day) were obtained from the FFQ and were assigned into 33 food groups (grams per day) based on a previous Australian study [24,40] for use in the dietary pattern analysis.

### 2.4. Dietary Pattern Analysis

Dietary patterns were derived using factor analysis with factor loadings extracted using the principal component method and varimax/orthogonal rotation. The number of dietary patterns identified was based on eigenvalues >1.5, on identification of a break point in the scree plot, and on interpretability [41]. Using these criteria, a 3-factor solution was chosen and rerun with the resulting factor scores saved and converted to *Z*-scores for analysis. Items with factor loadings ≥0.25 were considered as the items of relevance for the identified factor. These items represent the foods most highly related to the identified factor [42]. Foods that cross-loaded on several factors were retained.

### 2.5. Statistical Analyses

All statistical procedures were performed using SPSS version 22. Frequencies and descriptive statistics were expressed as *n* (%) and as means (SD), respectively. All reported *P* values were 2-tailed, and a *p*-value < 0.05 was considered to be statistically significant. Before hypothesis testing, data were examined for normality, in which all independent variables were normally distributed. Data were analysed by independent *t*–test to compare continuous variables and chi-square test to compare categorical variables between women with and without PCOS. Binary logistic regression analyses were used to test the association between PCOS (yes/no) and the independent variables for each dietary pattern (*Z*-score), with values presented as OR (95% CI). All logistic regression analyses were undertaken adjusting for potential confounders identified a priori, including maternal age, BMI, currently breastfeeding, number of children and waist circumference, or statistically, from association with PCOS on univariate analysis (*p* < 0.05). Multicollinearity was tested with binary regression analysis using the variance inflation factor (<5); no multicollinearity was observed between any of the independent variables. All model assumptions were validated with residual plots. Analyses were conducted using survey commands for analysing data weighted by area of residence to adjust for the deliberate over sampling in rural and remote areas.

## 3. Results

### 3.1. Participant Characteristics

Participant characteristics are reported in Table 1. The women with PCOS were around two months younger, were more likely to not have children and had a lower prevalence of currently breastfeeding compared to women without PCOS. As reported previously, women with PCOS also reported a higher BMI, weight and waist circumference than women without PCOS [7]. As previously reported [19], women with PCOS had an elevated energy and fibre intake and lower glycaemic index and percent energy intake from saturated fat compared to women without PCOS (data not reported). As previously reported [19], women with and without PCOS had similar physical activity levels (814 ± 874 *vs.* 820 ± 895 MET/min, *p* = 0.75).

**Table 1:** Characteristics for women with and without polycystic ovary syndrome.

	All *n* = 8200	PCOS *n* = 414	Non-PCOS *n* = 7155	*p*
Age (years) *	33.7 (1.5)	33.5 ± 0.1	33.7 ± 0.02	0.015
BMI (kg/m^2^) *	25.8 (5.9)	29.0 ± 0.4	25.4 ± 0.1	<0.001
Weight (kg) *	71.3 (16.7)	79.6 ± 1.2	70.3 ± 0.2	<0.001
Waist circumference (cm) *	86.0 (14.3)	91.9 ± 1.0	85.7 ± 0.2	<0.001
*Smoking status* †				0.729
Never smoker	4972 (60.4)	256 (59.1)	4341 (60.3)	
Ex-smoker	2112 (25.6)	121 (27.9)	1829 (25.6)	
Smoke <10 cigarettes/day	574 (6.9)	26 (6.0)	517 (7.1)	
Smoke 10–19 cigarettes/day	372 (4.5)	20 (4.6)	319 (4.4)	
Smoke ≥20 cigarettes/day	205 (2.5)	10 (2.3)	183 (2.5)	
*Personal income* †				0.765
No income	724 (9.5)	41 (9.8)	634 (9.1)	
Low (>$0–$36,399)	2923 (38.5)	156 (37.5)	2562 (36.3)	
Medium ($36,400–$77,999)	2737 (36.1)	137 (32.7)	2398 (34.0)	
High (>$78,000)	1207 (15.9)	71 (17.0)	1047 (14.9)	
*Highest qualification* †				0.762
No formal qual/year 10/12	1492 (18.4)	76 (18.1)	1301 (17.3)	
Equiv				
Trade/diploma	2040 (21.2)	100 (23.8)	1793 (23.9)	
Degree or higher	4565 (56.4)	245 (58.2)	3986 (53.1)	
*Marital status* †				0.630
Married	5115 (62.2)	260 (59.9)	4455 (62.0)	
De facto	1233 (15.0)	64 (14.7)	1067 (14.9)	
Separated/divorced	422 (5.1)	21 (4.8)	373 (5.2)	
Widowed	14 (0.2)	0 (0)	14 (0.2)	
Never married	1445 (17.6)	89 (20.5)	1274 (17.7)	
*Number of children* †				0.002
0	3134 (38.1)	205 (47.2)	2748 (36.1)	
1	1630 (19.8)	86 (19.8)	1409 (18.5)	
2–3	3228 (39.2)	132 (30.4)	2818 (37.0)	
≥4	243 (3.0)	11 (2.5)	213 (2.8)	
*Currently breastfeeding* †				0.003
No	4817 (58.4)	223 (51.3)	4193 (55.0)	
Yes	277 (3.4)	7 (1.6)	240 (3.2)	
No child	3149 (38.2)	205 (47.1)	2761 (36.2)	

* Values represent mean (SD); † Values represent *n* (%); Data were analysed by independent *t*-test to compare continuous variables and chi-square test to compare categorical variables between women with and without PCOS; BMI: Body mass index.

### 3.2. Dietary Patterns

The dietary pattern analysis revealed three distinct patterns explaining a total 27% variance (Table 2). The first pattern was labelled *Non-core foods* as there were high factor loadings for cakes, biscuits, sweet pastries; confectionary; refined grains and also take-away foods and crisps. The second pattern was labelled *High meat and take-away* as fish (fried, processed, canned and cooked), processed meat, red meat, but also take-away food highly correlated in this pattern. The final pattern was labelled *Mediterranean-style* as Mediterranean type foods highly correlated to this pattern including a variety of vegetables, fruit and nuts, small correlations with fish, while crisps were inversely correlated. Participant characteristics across the quartiles of dietary pattern score are presented in Supplemental Table 1.

**Table 2:** Factor loadings for each of the identified pre-conception dietary patterns for women with and without PCOS.

Food Group	Non-Core Foods	High Meat and Take-Away	Mediterranean-Style
Cakes, biscuits, sweet pastries	0.661	0.010	0.020
Confectionary	0.629	0.089	0.020
Refined grains	0.483	0.239	0.146
Vegemite	0.483	0.106	0.068
Takeaway	0.467	0.402	−0.138
Crisps	0.466	0.199	−0.262
Juice	0.408	0.007	0.071
Tomato sauce	0.380	0.018	0.029
Processed meat	0.359	0.567	−0.190
Red meat	0.330	0.595	−0.088
Added sugar	0.325	−0.023	−0.120
Wholegrains	0.319	−0.113	0.408
Saturated spreads	0.291	−0.117	0.111
Poultry	0.280	0.520	−0.098
Potato	0.279	0.009	−0.199
Nuts and nut spread	0.260	0.137	0.493
Fried fish	0.212	0.649	−0.064
Fresh fruit	0.150	−0.020	0.539
Tomatoes	0.081	−0.137	0.355
Legumes	0.066	0.018	0.207
Other vegetables	−0.008	0.114	0.618
Leafy green vegetables	−0.038	0.082	0.503
Eggs	−0.039	0.202	0.271
Processed fish	−0.040	0.510	0.376
Other fish	−0.042	0.620	0.260
Garlic	−0.055	0.033	0.435
Soya	−0.082	−0.040	0.393
Alcohol	−0.185	0.287	0.060
*Percentage variance explained*	13%	8%	6%

Dietary patterns obtained using factor analysis with factor loadings extracted using the principal component method and varimax/orthogonal rotation. Food groups with factor loadings <0.25 for all factors are not included in the table (cruciferous vegetables; yellow or red vegetables; low fat dairy; full fat dairy; and canned fruit).

### 3.3. Dietary Patterns and PCOS

Table 3 reports the results from logistic regression. In the crude analysis, for each 1 SD increase in the *High meat, fish, poultry and take-away* pattern, there was a 9% greater likelihood for women to have PCOS, however this association did not remain in the adjusted analysis. In the crude analysis, for each 1 SD increase in the *Mediterranean-style* dietary pattern, there was a 15% greater likelihood for women to have PCOS. This association was strengthened after adjusting for maternal age, maternal BMI, current breastfeeding, number of children, such that for each 1 SD increase in the *Mediterranean-style* dietary pattern, there was a 26% greater likelihood that the women reported had PCOS. There were no associations between the *Unhealthy, non-core foods* pattern and PCOS.

**Table 3:** Odds ratios for likelihood of PCOS according to the dietary patterns identified.

	OR *	95% CI	*p*	Adjusted OR * †	95% CI	*p*
PCOS						
Unhealthy, non-core foods	1.06	0.97, 1.16	0.22	1.03	0.94, 1.13	0.55
High meat, fish, poultry and take-away	1.09	1.00, 1.17	0.03	1.04	0.95, 1.13	0.43
Mediterranean-style	1.15	1.05, 1.26	0.02	1.26	1.15, 1.39	<0.001
Maternal age	-			0.92	0.85, 0.98	0.014
Maternal BMI	-			1.09	1.07, 1.10	<0.001
Current breastfeeding	-			1.00	0.96, 1.05	0.97
Number of children	-			0.88	0.75, 1.04	0.13

Associations between PCOS and dietary patterns (*Z*-scores) in crude and adjusted analyses carried out using binary logistic regression analyses; Referent category is not having PCOS; * Indicates change in risk per 1 SD increase in factor score; **†** Adjusted for maternal age, maternal BMI, current breastfeeding, number of children.

## 4. Discussion

We report here for the first time that women with PCOS have different dietary patterns compared to women without PCOS, in a large population-based cohort of women. Women with PCOS were more likely to consume a dietary pattern consistent with the Mediterranean diet; however there were no differences in the other commonly consumed dietary patterns of unhealthy non-core foods or a pattern higher in meat.

The Mediterranean-style dietary pattern contains a number of foods similar to a Mediterranean diet which consists of fish, monounsaturated fats from olive oil, fruits, vegetables, wholegrains, legumes and nuts and moderate alcohol consumption [43]. It is also consistent with previously defined “Mediterranean” patterns in prior research comprising vegetables, fish, fruits, poultry, low-fat dairy products, and olive oil [44,45]. Surprisingly however, we found an inverse factor loading for poultry which is typically consumed in higher intakes in the Australian population compared to fish [46], which loaded on this pattern in moderate amounts for both processed (*i.e.*, tinned fish) and other fish (*i.e.*, cooked fish), while fried fish was inversely associated. It is to be noted that intake of olive oil is not collected in the food frequency questionnaire used in this study. Another surprising finding was that both low fat and high fat dairy foods did not correlate to any pattern. This might reflect the overall low consumption of dairy in men and women in the adult Australian population; yet this is consistent with a previous study in pregnant women where low fat dairy did not load on any of the three dietary patterns, and high fat dairy only moderately correlated with the vegetarian-type dietary pattern [24]. Nevertheless, non-core foods inversely loaded on this pattern such as take-away foods and crisps, as well as added sugar, which supports an overall healthier dietary pattern consisting of a number of Mediterranean foods. As we are the first to report that a Mediterranean-style dietary pattern was independently associated with increased likelihood of having PCOS, this discrepant finding may indicate the possible high level of women with PCOS seeking dietary knowledge with a subsequent adoption of healthy dietary patterns.

To date, there are few other studies reporting on the relationship between dietary patterns and other conditions co-existing with PCOS. In literature assessing infertile women, a large proportion of whom will likely have PCOS, a Mediterranean diet is associated with a higher chance of natural or assisted reproduction conception [44,47]. The adoption of a Mediterranean-style diet in PCOS may therefore have positive implications for the appropriate lifestyle management of chronic diseases associated with PCOS. Further studies are needed to expand on our findings on the association of dietary changes in those with a diagnosis of PCOS, the optimal means of conveying dietary education at diagnosis and the long-term maintenance of positive dietary changes.

We observed here that the two other identified dietary patterns, namely those consisting predominantly of non-core foods or a higher meat intake from either take-away/processed or non-processed sources explained a moderate proportion of variability in food intake in all participants (13% and 8% respectively). However, neither pattern was associated with PCOS status in the adjusted analysis. In association with higher weight and BMI in PCOS, this is a positive finding that is also consistent with the diagnosis of PCOS contributing to an improvement of dietary habits in keeping with population-based dietary guidelines of minimising discretionary or non-core food intake, reducing processed meats and consuming a moderate intake of protein [48].

While a Mediterranean diet is not a specifically recommended dietary intake for PCOS, emerging research suggests beneficial effects of certain components of this diet, such as elevated omega-3 fatty acids which are generally found in high amounts of oily fish. Although the specific types of fish consumed in our Mediterranean style dietary pattern cannot be extracted, both processed fish and cooked fish varieties contain some omega-3 fatty acids, likely contributing to a reasonable intake of omega-3 fatty acids in this population. The literature in PCOS focuses predominantly on omega-3 fatty acid supplementation studies which report improvements in outcomes including reductions in bioavailable androgens, triglycerides, blood pressure, glucose and surrogate markers of insulin resistance [49,50,51,52]. One recent study found that a Mediterranean diet pre-pregnancy was associated with a 42% reduced likelihood of developing hypertensive related disorders during pregnancy [26]; while higher consumption of sweets and seafood [25] or high intake of red meat, processed meat, refined grain products and sweets [27] during pregnancy was associated with a 23% and 63% increased risk of gestational diabetes. A Mediterranean dietary pattern has also been reported to be associated with improved health outcomes including decreased inflammation [53] and prevalence of the metabolic syndrome [54], abnormal glucose tolerance [55] or depression [45]. As adverse health outcomes are commonly associated with PCOS [3,4,6], this dietary pattern may therefore result in health benefits. However, we have previously reported in this cohort that this improved diet quality occurred in conjunction with a modest increase in energy intake (+215 kJ/day) which could contribute to additional longitudinal weight gain [19]. The potential benefits of an improved dietary pattern may not outweigh the effects of increased energy intake and consequent weight gain with regards to effects on reproductive, and potentially metabolic and psychological, parameters.

Strengths to our study include the large population of women with and without PCOS from a community-based population in contrast to the majority of the existing research assessing diet and PCOS. This minimises selection bias. This is also more likely to capture a lower proportion of women with PCOS with a more severe clinical phenotype and a higher BMI who typically present to clinical services and are captured in research studies [56]. While the use of self-report PCOS is a limitation, the nature of this research means that it is not feasible to clinically verify PCOS or control status. It is also not possible to determine the PCOS phenotype or which diagnostic criteria were used in diagnosis. However, given that the Rotterdam criteria were first published in 2004 [57], it is also most likely that the majority of women self-reporting diagnosed PCOS in Survey 4, conducted in 2006, would have been diagnosed based on NIH criteria. There are also some other limitations to our study. We report here 58% participant retention compared to baseline levels 13 years prior which may indicate bias and limit generalisability. However, no differences between completors and non-completors has previously been reported indicating a likely minimal effect of attrition on outcomes [36]. Although the FFQ is a validated measure of assessing nutritional intake, we are not able to assess the contribution of dietary patterns to the development or severity of PCOS due to the study design and report here only associations between dietary patterns and PCOS status. Further, the total variance explained by each factor was intermediate compared with previous factor analyses conducted in different age groups [29,58,59]; however, the Kaiser-Meyer-Olkin measure of sampling adequacy was 0.78, exceeding the recommended value of 0.6; and Bartlett's test of Sphericity achieved statistical significance indicating the correlations in the data set are appropriate for factor analysis. Moreover, the food groups loading on the factors were varied and many were greater than the 0.25 cut-off value suggesting that our population had a varied diet that was, nevertheless, still specific to the identified factors. As the present study is the first of its kind in this population, further studies are required to refute or support our findings and future work is warranted assessing the contribution of dietary pattern intake to the severity or incidence of PCOS.

## 5. Conclusions

In conclusion, we report for the first time the independent association of PCOS status with self-reported dietary patterns, specifically a Mediterranean diet pattern. This may indicate an improvement in the quality of dietary intake following a diagnosis of PCOS. We also report no increase in dietary patterns high in non-core, meat or take-away foods despite a higher body weight. Combined with our prior research showing healthier intake but higher caloric consumption, it appears that women with PCOS may have a greater appetite and are more overweight, despite a healthier diet. Future research should examine the contribution of dietary patterns to the incidence and severity of PCOS and the potential for modification of dietary patterns in the lifestyle management of PCOS.

## Supplementary Files

Supplementary File 1
