# Supplementary material for: The Association of a Mediterranean-Style Diet Pattern with Polycystic Ovary Syndrome Status in a Community Cohort Study
Source: Nutrients. 2015 Oct 16;7(10):8553–64. doi: 10.3390/nu7105419 (PMC4632439; doi:10.3390/nu7105419)
Supplement: Supplementary File 1 [file nutrients-07-05419-s001.docx]

**Table S1.** Characteristics for women with and without polycystic ovary syndrome, according to dietary pattern quartile

|  | **Non-core foods** | | | | **High meat and take-away** | | | | **Mediterranean-style** | | | | |
| --- | --- | --- | --- | --- | --- | --- | --- | --- | --- | --- | --- | --- | --- |
| *No PCOS / PCOS* | **Q1** | **Q2** | **Q3** | **Q4** | **Q1** | **Q2** | **Q3** | **Q4** | **Q1** | **Q2** | **Q3** | **Q4** |  |
| Age (years)* | 33.7 / 33.6 | 33.7 / 33.3 | 33.7 / 33.6 | 33.7 / 33.4 | 33.7 / 33.4 | 33.7 / 33.5 | 33.7 / 33.6 | 33.7 / 33.4 | 33.8 / 33.4 | 33.6 / 33.5 | 33.7 / 33.4 | 33.7 / 33.7 |  |
| BMI (kg/m^2^)* | 25.5 / 29.1 | 25.5 / 28.4 | 25.3 / 28.9 | 26.5 / 30.5 | 24.5 / 28.9 | 25.4 / 28.2 | 26.0 / 28.8 | 26.9 / 30.5 | 26.8 / 31.4 | 26.1 / 29.5 | 25.5 / 28.1 | 24.4 / 28.4 |  |
| Weight (kg)* | 70.1 / 80.0 | 70.1 / 76.9 | 70.4 / 79.2 | 73.1 / 84.8 | 67.4 / 78.6 | 70.2 / 77.9 | 71.8 / 78.1 | 74.4 / 85.2 | 73.5 / 85.4 | 71.9 / 81.4 | 70.3 / 77.0 | 67.9 / 79.2 |  |
| Waist circumference (cm)* | 84.8 / 91.5 | 85.4 / 90.5 | 85.9 / 91.3 | 88.8 / 96.0 | 84.3 / 91.7 | 85.6 / 89.7 | 86.8 / 90.1 | 88.4 / 95.8 | 89.1 / 96.9 | 87.4 / 94.5 | 85.4 / 90.8 | 83.3 / 89.1 |  |
| *Smoking status*† |  |  |  |  |  |  |  |  |  |  |  |  |  |
| Never smoker | 22 / 26 | 26 / 23 | 26 / 21 | 26 / 29 | 29 / 26 | 27 / 23 | 24 / 23 | 20 / 28 | 23 / 21 | 25 / 23 | 26 / 27 | 25 / 28 |  |
| Ex-smoker | 27 / 26 | 24 / 25 | 26 / 20 | 24 / 19 | 21 / 20 | 24 / 22 | 27 / 17 | 27 / 41 | 24 / 19 | 23 / 17 | 25 / 29 | 28 / 35 |  |
| Smoke <10 cigarettes/d | 29 / 27 | 26 / 36 | 23 / 9 | 23 / 27 | 20 / 27 | 25 / 18 | 25 / 18 | 29 / 36 | 23 / 9 | 26 / 0 | 20 / 55 | 31 / 36 |  |
| Smoke 10-19 cigarettes/d | 45 / 50 | 24 / 0 | 18 / 0 | 14 / 50 | 17 / 50 | 14 / 0 | 26 / 25 | 43 / 25 | 25 / 0 | 29 / 50 | 20 / 25 | 26 / 25 |  |
| Smoke ≥20 cigarettes/d | 32 / 30 | 23 / 26 | 21 / 16 | 24 / 28 | 17 / 19 | 20 / 12 | 25 / 42 | 38 / 28 | 39 / 49 | 27 / 21 | 20 / 12 | 14 / 19 |  |
| *Personal income*† |  |  |  |  |  |  |  |  |  |  |  |  |  |
| No income | 16 / 18 | 23 / 23 | 27 / 28 | 33 / 33 | 29 / 20 | 27 / 23 | 23 / 25 | 8 / 33 | 22 / 40 | 26 / 13 | 28 / 28 | 24 / 20 |  |
| Low (>$0–$36,399) | 19 / 18 | 24 / 21 | 28 / 24 | 30 / 37 | 27 / 24 | 25 / 20 | 26 / 27 | 23 / 29 | 30 / 25 | 25 / 21 | 23 / 24 | 23 / 29 |  |
| Medium ($36,400–$77,999) | 27 / 34 | 25 / 20 | 25 / 18 | 23 / 28 | 24 / 22 | 25 / 24 | 27 / 22 | 24 / 33 | 24 / 26 | 24 / 20 | 27 / 31 | 26 / 23 |  |
| High (>$78,000) | 32 / 32 | 31 / 24 | 24 / 21 | 14 / 23 | 22 / 21 | 29 / 20 | 22 / 20 | 27 / 38 | 15 / 9 | 27 / 9 | 27 / 21 | 31 / 44 |  |
| *Highest qualification*† |  |  |  |  |  |  |  |  |  |  |  |  |  |
| No formal qualification/year 10/12 equivalent | 21 / 30 | 27 / 20 | 26 / 19 | 27 / 35 | 25 / 24 | 21 / 17 | 23 / 28 | 30 / 31 | 47 / 33 | 23 / 30 | 19 / 13 | 15 / 24 |  |
| Trade/diploma | 26 / 27 | 25 / 34 | 24 / 16 | 25 / 23 | 22 / 24 | 24 / 11 | 25 / 27 | 31 / 41 | 31 / 29 | 26 / 29 | 27 / 15 | 25 / 30 |  |
| Degree or higher | 24 / 29 | 26 / 25 | 26 / 21 | 24 / 26 | 25 / 27 | 26 / 25 | 25 / 22 | 20 / 26 | 16 / 13 | 24 / 19 | 28 / 35 | 33 / 34 |  |
| *Marital status*† |  |  |  |  |  |  |  |  |  |  |  |  |  |
| Married | 21 / 19 | 25 / 26 | 27 / 22 | 27 / 33 | 27 / 23 | 27 / 23 | 25 / 26 | 22 / 29 | 25 / 23 | 26 / 25 | 26 / 30 | 23 / 23 |  |
| De facto | 26 / 25 | 24 / 20 | 25 / 16 | 25 / 23 | 24 / 30 | 22 / 21 | 28 / 21 | 27 / 28 | 25 / 31 | 24 / 8 | 24 / 16 | 28 / 44 |  |
| Separated/divorced | 3838 | 23 / 15 | 21 / 15 | 19 / 32 | 24 / 13 | 23 / 21 | 18 / 30 | 34 / 36 | 33 / 27 | 24 / 9 | 20 / 27 | 22 / 38 |  |
| Widowed | 42 / 0 | 16 / 0 | 37 / 0 | 5 / 0 | 26 / 0 | 26 / 0 | 16 / 0 | 32 / 0 | 37 / 0 | 37 / 0 | 21 / 0 | 5 / 0 |  |
| Never married | 34 / 36 | 26 / 25 | 21 / 17 | 19 /22 | 23 / 25 | 24 / 16 | 24 / 18 | 29 / 41 | 22 / 18 | 23 / 23 | 25 / 23 | 30 / 36 |  |
| *Number of children*† |  |  |  |  |  |  |  |  |  |  |  |  |  |
| 0 | 34 / 35 | 26 / 27 | 22 / 17 | 18 / 22 | 24 / 27 | 25 / 17 | 26 / 20 | 26 / 36 | 18 / 16 | 24 / 21 | 16 / 28 | 32 / 35 |  |
| 1 | 19 / 25 | 25 / 21 | 27 / 25 | 29 / 30 | 31/ 14 | 24 / 28 | 24 / 25 | 21 / 33 | 24 / 28 | 25 / 20 | 25 / 25 | 26 / 27 |  |
| 2-3 | 20 / 19 | 24 / 25 | 27 / 19 | 28 / 37 | 25 / 26 | 26 / 21 | 25 / 26 | 23 / 29 | 32 / 27 | 25 / 31 | 25 / 22 | 18 / 20 |  |
| ≥4 | 11 / 44 | 17 / 3 | 36 / 31 | 36 / 22 | 19 / 28 | 19 / 50 | 15 / 11 | 50 / 8 | 36 / 61 | 39 / 6 | 11 / 25 | 15 / 8 |  |
| *Currently breastfeeding*† |  |  |  |  |  |  |  |  |  |  |  |  |  |
| No | 20 / 21 | 25 / 22 | 27 / 22 | 28 / 36 | 26 / 20 | 25 / 24 | 25 / 27 | 24 / 20 | 30 / 30 | 26 / 21 | 24 / 25 | 20 / 45 |  |
| Yes | 9 / 0 | 19 / 40 | 25 / 20 | 48 / 40 | 35 / 60 | 27 / 40 | 22 / 0 | 16 / 0 | 17 / 0 | 20 / 60 | 28 / 40 | 35 / 0 |  |
| No child | 34 / 35 | 26 / 27 | 22 / 17 | 18 / 22 | 24 / 27 | 25 / 17 | 25 / 20 | 26 / 36 | 18 / 16 | 24 / 21 | 26 / 28 | 32 / 35 |  |

*Values represent mean; † Values represent %
